# Combining stated and revealed preferences to define smallholder cattle breeding objectives in northwest Ethiopia: Evidence for breeding program design

**DOI:** 10.1371/journal.pone.0359944

**Published:** 2026-10-07

**Authors:** Abiy Shenkut Abebe, Belete Yazie, Melaku Menale

**Affiliations:** Department of Animal Science, Debre Tabor University, Debre Tabor, Ethiopia; Universidade Federal de Mato Grosso do Sul, BRAZIL

## Abstract

Identifying high-priority traits is essential for designing effective breeding programs in smallholder systems. This study employed both stated and revealed preference methods to identify priority traits for cattle breeding objectives among smallholders in northwest Ethiopia. Stated preferences were assessed using a discrete choice experiment (DCE), while revealed preferences were examined through a hedonic pricing approach. The two datasets were analyzed separately and their results compared to provide complementary insights. Data were collected between January and April 2024: 384 farmers participated in the choice survey across three districts (Farta = 91, Simada = 133, and Lay Gayint = 160), and 200 cattle transactions were recorded in three marketplaces—Debre Tabor, Simada, and Nefas Mewucha. Choice data were analyzed with a mixed logit model, and market data with a log-linear ordinary least squares regression. Results showed both convergence and divergence in trait preferences between methods. Farmers’ strong preference for large body size in the DCE (p < 0.001) aligned with the significant positive effects of body condition and live body weight on market price (p < 0.001). However, traits not visually observable at sale—such as milk yield, docility, disease tolerance, libido, calving interval, and calf vigor—were highly valued in stated preferences but not captured in market prices due to observability constraints and information asymmetry. Conversely, the hedonic model revealed market structure effects not captured by the DCE, including higher prices paid by traders and premiums for slaughter animals. These findings demonstrate that employing both methods provides a more comprehensive evidence base for defining breeding objectives—capturing both invisible livelihood-critical traits valued by farmers and market dynamics that shape real-world prices. The identified priority traits provide a foundation for subsequent research to estimate genetic parameters and economic weights to design a breeding program.

## Introduction

Cattle are the primary livestock species in Ethiopia, supporting the livelihoods of millions of smallholder farmers. They provide essential draft power for rural communities and are valued for their meat, milk, organic fertilizer, and cash income [1–3]. With an estimated 70.9 million head, Ethiopia has the largest cattle population in Africa and ranks fifth worldwide [4]. Indigenous breeds constitute 97.4% of the national herd, while crossbreds and exotic breeds account for the remainder. Although the cattle population has grown significantly over the past decade [4], the productivity of indigenous cattle remains low. Given that livestock—particularly cattle—are major contributors to agricultural greenhouse gas emissions, maintaining fewer but more productive animals is critical for supporting smallholder livelihoods, fostering national economic growth, and mitigating climate change.

To enhance productivity, crossbreeding with exotic breeds such as Holstein Friesian and Jersey has been practiced for decades [5,6]. Yet, despite these efforts, performance remains limited. For instance, during a typical seven-month lactation period, cows produce an average of only 1.5 liters of milk per day [7]. Consequently, annual per capita milk consumption in Ethiopia is about 19 liters—far lower than in neighboring Uganda (50 liters) and Kenya (90 liters) [8], and well below the World Health Organization’s recommended intake of 200 liters per year. In addition to low milk yield, indigenous cattle face reproductive challenges, including extended days open, late age at first calving, long calving intervals, and short lactation lengths [7,9].

Despite these constraints, Ethiopian indigenous cattle exhibit considerable within-breed genetic diversity [10,11], highlighting strong potential for genetic improvement through selective breeding. They are also well adapted to the low-input production systems typical of northwest Ethiopia [12]. However, systematic selective breeding programs that leverage this adaptive potential and genetic diversity are virtually absent, particularly in the northwest. A critical step in designing such programs is defining the breeding objective—identifying the specific traits to be improved through selection [13,14]. Yet, up-to-date information on smallholder breeding objectives for indigenous cattle in northwest Ethiopia is lacking. Moreover, given Ethiopia’s diverse agro-ecologies, community groups, and livestock production systems, smallholders’ preferences for cattle traits are likely to vary across contexts.

At the smallholder level, participatory methods such as discrete choice experiments (DCEs) and hedonic pricing have increasingly been used to identify livestock trait preferences in Ethiopia [12,15,16] and elsewhere [17,18]. A DCE is a stated preference method in which individuals reveal preferences through hypothetical choice sets, while hedonic pricing is a revealed preference method that infers preferences from actual market transactions.

Both approaches have strengths and limitations [19,20]. DCEs are prone to hypothetical bias, arising from inconsistencies between stated and actual behavior, whereas hedonic pricing often suffers from endogeneity bias due to omitted variables and limited purchasing choices. Importantly, the limitations of one method are not necessarily constraints for the other. This complementarity suggests that combining both methods can harness their respective strengths while mitigating weaknesses. Accordingly, the present study employed both DCE and hedonic pricing to define smallholder cattle breeding objectives in northwest Ethiopia. The findings aim to inform the design of a conservation-based breeding program for indigenous cattle.

## Materials and methods

### Ethics approval statement

This study was reviewed and approved by the Ethical Review Committee of Debre Tabor University (reference number: Ref: DTU/CAES/RCC/08/115/24). The study did not involve any experimental or clinical trial on live animals. The survey and data handling followed the required ethical guidelines and were carried out from January 5 to April 30/2024. Participants were first informed about the purpose of the study, after which informed consent was obtained from all participants prior to the interview. Participation was entirely voluntary, and all participants were 18 years of age or older. No sensitive information (health status, religious beliefs, political views, or illegal behavior) was collected, and all data were anonymized. Morphometric data (heart girth and body length) were collected non-invasively from 200 traded cattle using a flexible measuring tape in an open market, ensuring animal welfare and causing no stress.

### Description of the study areas

This study was conducted in the Farta, Simada, and Lay Gayint districts of the South Gondar Zone, located in northwest Ethiopia. These districts were selected because they host relatively large cattle populations compared to other districts in the zone and represent the natural habitat of the Simada cattle breed. The study aimed to investigate smallholders’ trait preferences to generate inputs for designing a conservation-based genetic improvement program for this breed.

Agricultural practices in the study districts are characterized by mixed crop–livestock farming systems, with cattle serving as the principal source of draft power for cropland preparation [12]. Agro-ecologically, Farta and Lay Gayint are predominantly highland areas, while Simada is mainly mid-altitude. According to the Central Statistical Agency of Ethiopia (CSA) [21], the South Gondar Zone has approximately 1.9 million cattle, with the three study districts collectively accounting for about one-third of this population. Climatic data—including average daily minimum and maximum temperatures and annual rainfall—for the South Gondar Zone and the individual study districts are summarized in Table 1.

**Table 1:** Climate variables in the study areas.

Climate variables^#^	South Gondar zone	Study districts			Sources
		Farta	Simada	Lay Gayint	
Average annual rainfall (mm)	1307	1552	1144	1132	[22]
Mean annual minimum temperature (°C)	13.76	11.7	14	10.1	[23–25]
Mean annual maximum temperature (°C)	25.1	24	25	21.6	

#Rainfall data from 2020/01/01 to 2025/01/01 were taken from CHIRPS-daily, while temperature data from 2010/05/01 to 2016/05/01 were generated from CHIRTS-daily.

### Study approach

This study employed both DCE and hedonic pricing methods to examine smallholder preferences for indigenous cattle traits. The target population for both methods was smallholder farmers in the selected districts. Choice data for the DCE were collected through a cross-sectional survey administered to randomly selected farmers, while transaction data for the hedonic pricing method were obtained by observing cattle sales in the major markets of the study districts. Although both methods focused on the same target population, the specific respondents and data sources differed. Consequently, the datasets were analyzed separately and independently rather than merged, and their results were subsequently compared and integrated to provide complementary insights. This study therefore represents a comparative and complementary integration of evidence, not a statistical integration of the two datasets.

### Experimental design and data collection

#### Stated preference data

A key step in designing a DCE is the identification of relevant attributes and their levels [12,26]. For this study, appropriate cattle attributes were identified through a combination of methods, including Participatory Rural Appraisal (PRA) and consultations with researchers and livestock experts. In each study district, PRA was conducted in the form of focus group discussions with key informants and livestock experts. The key informants were experienced farmers with in-depth knowledge of the socio-economic and cultural values of indigenous cattle, identified with the assistance of district livestock experts.

During the discussions, various cattle attributes were listed and prioritized using a pairwise ranking method. The six highest-ranking attributes for bulls and the six for cows were selected for the choice design. In addition, market price was included as a seventh attribute for both bulls and cows, given its importance in determining farmers’ marginal willingness to pay (MWTP) for genetic improvements in other traits. The list of attributes, along with their levels and descriptions, is presented in Table 2.

**Table 2:** Bull and cow attributes with their descriptions and effect coded levels.

Attributes	Attribute descriptions	Levels with effect-coded
Body size	Physical appearances including the height and body length of a bull or a cow	Large = 1 Small = −1
Draft power strength^$^	Bull’s endurance for plowing cropland per day	Strong = 1, if able to plow for 8 hrs. Weak = −1, if plow for 4 hrs., or less
Disease resistance	The frequency of visiting a veterinary clinic per year due to illness of a bull or a cow	Tolerant = 1, if never gets illness Susceptible = −1, if gets ill at least once per year
Temperament	Behavioral reactions of a bull or a cow observed during handling	Aggressive = 1 Docile = −1
Color^$^	The type of coat color predominantly observed on the body of a bull or a cow	Plain = 1 Mixed = −1
Libido^$^	A bull's willingness and eagerness to attempt mounting and successfully service a cow in heat	Active = 1 In-active = −1
Milk yield^#^	Milk yield of a cow per day in liter^&^	High = 1, if daily milk yield is greater than 5 litters Low = −1, if daily milk yield is less than 2 litters
Calf vigor^#^	Strength of newborn calf to quickly stand and suckle enough colostrum to achieve the protection of passive immunity	Strong = 1, if sit up within 15 minute after birth and stand and suckle enough colostrum within four hours without assistance Weak = − 1, if it requires human assistance
Calving interval^#^	The time between two successive calving in the same cow.	Short = 1, if the interval is one year Long = −1, if the interval is greater than one year
Purchasing price*	Purchasing price of a four-year-old mature bull	50, 000 ETB 40,000 ETB 30,000 ETB
	Purchasing price of a four-year-old mature cow in Ethiopian Birr (ETB)	40, 000 ETB 30,000 ETB 20,000 ETB

^$^ Bull attributes only, ^#^cow attributes only and unmarked attributes appeared in both bull and cow choice sets

^*^Between January and April 2024, the average exchange rate was ~ 56.35 ETB per 1 USD.

^&^ Milk yield thresholds based on Haile et al. [5] for crossbreds (>5 L/day) and Mekuriaw & Harris-Coble [7] for indigenous cattle (<2 L/day).

Employing a full factorial design consisting of six attributes at two levels and one attribute at three levels $\left({\text{2}}^{\text{6}}\;X\;{\text{3}}^{\text{1}}\right)$ yielded 192 possible choice profiles. However, presenting all combinations was impractical for smallholder respondents. Therefore, a fractional factorial design was used to reduce the number of alternatives. Sixteen choice alternatives, categorized into four blocks, were generated using JMP statistical discovery software [27]. The design achieved a D-efficiency score of 98.85, indicating high efficiency in estimating the effects of the study variables. To avoid forced choices, an opt-out option was included in each block. Thus, each block contained five alternatives for both bulls and cows, with respondents required to make a single choice decision per block.

Pictures were used to illustrate attribute levels for both bulls and cows. A sample choice card for a bull is presented in Fig 1. The choice survey was conducted between January and April 2024 by trained enumerators under close supervision of researchers. A total of 384 smallholder farmers randomly selected from the three districts (Farta = 91, Simada = 133, Lay Gayint = 160) participated. Farmers were first briefed on the study objectives and given sufficient time to understand the hypothetical choice alternatives. Choice cards from block one were introduced first, with participants proceeding sequentially through the remaining three blocks after selecting the most preferred alternative in each preceding task. Although an opt-out option was available, none of the participants selected it, suggesting that the presented alternatives were plausible and realistic.

**Fig 1:**
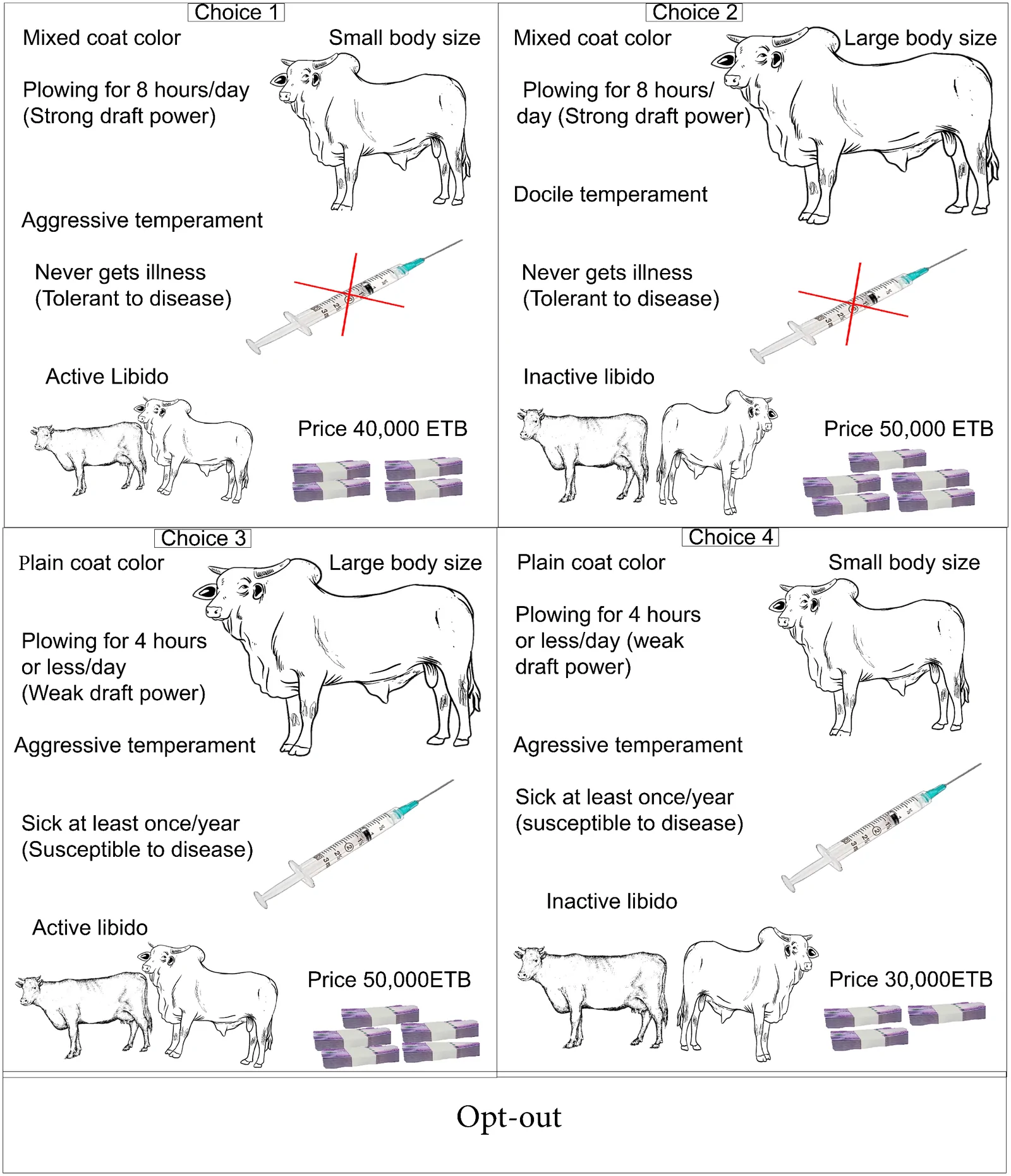
A sample choice card for a bull, presented to respondents.

The zero selection rate for the opt-out alternative, while uncommon in consumer-market choice experiments, is both behaviorally rational and methodologically documented within smallholder farming systems. For instance, Abebe et al. [12] reported an opt-out frequency below 0.5% for sheep traits, while Kassie et al. [28] observed a 1.4% opt-out rate for indigenous cow traits in Ethiopia. Because the explicit goal of the choice task was to define the breeding objective, the opt-out alternative was framed to farmers as: “no genetic improvement or trait modification is needed for your herd relative to the presented options.” Given the subsistence constraints of the crop-livestock production system, selecting the status quo was viewed as unviable by the respondents, illustrating how local context dictates choice behavior. Consequently, due to this complete lack of statistical variation, the opt-out alternative was excluded from the final econometric estimation matrix to ensure model convergence.

### Revealed preference data

Cattle transaction data were collected between January and April 2024 from three major marketplaces—Debre Tabor, Simada, and Nefas Mewucha—which represent the Farta, Simada, and Lay Gayint districts, respectively. In Ethiopia, the absence of a standardized livestock marketing system means that prices are determined through mutual agreement between sellers and buyers. Under such conditions, transaction prices are expected to be influenced by multiple factors.

To identify cattle price determinants, data were collected on buyers’ characteristics, purchasing purpose, and several observable cattle attributes, including sale price (see Table 3). These data were recorded only after the seller and buyer had reached full agreement, without their prior knowledge of the data collection, to avoid influencing negotiations. During data collection, cattle age was determined using dentition, and only animals with at least one pair of well-developed permanent incisors were included. In total, market data for 200 cattle transactions were collected. Of these, 56 transactions were observed in the Simada market, 69 in Debre Tabor, and 75 in Nefas Mewucha. The market transaction dataset includes 10 categorical and one continuous variables, with 11 predictors in total. According to Harrell [29], the ratio of sample size (m) to number of predictors (p) should satisfy p < m/10 (liberal), p < m/15 (good average), or p < m/20 (conservative). With m = 200 and p = 11, our study satisfies both the liberal rule (11 < 20) and the good average rule (11 < 13.33), implying the sample size was reasonably adequate to estimate the effects of 11 predictors without introducing overfitting. Achieving a higher sample size is often difficult in field-based livestock market studies, especially in the study area where standardized livestock marketing is absent.

**Table 3:** Revealed preference variables description with mean and standard deviations.

Variable	Variable description	Mean	Std. dev.
Price	Price per head of marketed cattle (ETB)	38127.20	11140.90
Market place	Live animal marketplace (Debre Tabor = base category)		
Nefas Mewucha	1 if Nefas Mewucha, 0 otherwise	0.38	0.49
Simada	1 if Simada, 0 otherwise	0.28	0.45
Education	Buyer education (elementary school = base category)		
Read and write	1 if the buyer can read & write, 0 otherwise	0.50	0.50
Illiterate	1 if illiterate, 0 otherwise	0.21	0.40
Buyer type	Buyer type (farmer = base category)		
Trader	1 if buyer type is trader, 0 otherwise	0.45	0.50
Purpose of buying	Purpose of buying cattle (ploughing = base category		
Breeding	1 if the purpose is for breeding, 0 otherwise	0.15	0.35
Resell	1 if the purpose is for resell, 0 otherwise	0.30	0.46
Slaughter	1 if the purpose is for slaughter, 0 otherwise	0.31	0.46
Horn status	Horn status of the cattle (polled = base category)		
Horned	1 if a cattle is horned, 0 otherwise	0.87	0.34
Sex of the cattle	Sex of marketed cattle (female = base category)		
Male	1 if the sex of the cattle is male, 0 otherwise	0.79	0.41
Breed of cattle	Cattle breed (crossbred = base category)		
Local	1 if local breed, 0 otherwise	0.77	0.43
Age of cattle*	The dentition of marketed cattle (3PPI = base category)		
1PPI	1 if a marketed cattle has 1PPI, 0 otherwise	0.17	0.38
2PPI	1 if a marketed cattle has 2PPI, 0 otherwise	0.30	0.46
Full mouth	1 if a marketed cattle has 4PPI, 0 otherwise	0.19	0.39
Coat color	Cattle coat color (mixed = base category)		
Brown	1 if the coat color is brown, 0 otherwise	0.23	0.42
Black	1 if the coat color is black, 0 otherwise	0.43	0.50
Body condition^#^	Body condition of cattle (moderate = base category)		
Good	1 if body condition is good, 0 otherwise	0.33	0.47
Live body weight^$^	Predicted live body weight in kg	308.03	95.80

*PPI – pair of permanent incisors, where 1PPI ~ 2 years-old, 2PPI ~ 3 years-old, 3PPI ~ 4 years-old and full mouth representing cattle with over 5 years of age).

^#^ Body condition was scored as moderate if ribs are slightly visible with some fat under the skin, while it was considered as good if ribs and pin bones are not visible unless touched with pressure.

^$^ Live body weight (BW) was predicted using Schaeffer's formula:$BW\;(kg)=\left(\frac{BL*{HG}^{\text{2}}}{\text{3}\text{0}\text{0}}\right)*\text{0}.\text{4}\text{5}\text{3}\text{6}$, where BL is body length of the animal measured from point of shoulder to pin bone in inches and HG is the chest girth of the animal in inches.

### Statistical modeling

#### Stated preference.

Choice data were analyzed using a mixed logit model implemented in the STATA software package [30]. The model is grounded in random utility theory, which posits that an individual’s choices can be modeled as a function of the attributes that define the available options [31]. According to this theory, selecting one option over others indicates that the chosen option provides higher utility [32]. In this study, smallholders are assumed to maximize their utility by selecting their most preferred alternatives for breeding bulls and cows.

The mixed logit model accounts for correlations among choices by incorporating random parameters [26]. A key advantage of this model is that it does not require the Independence of Irrelevant Alternatives (IIA) assumption, which is a limitation of the standard multinomial logit model. In the mixed logit framework, the utility ($U_{ij}$) for smallholder i deriving from alternative j can be expressed by the following equation:


$$\begin{array}{c} U_{ij}=\;\beta_{i}X_{ij}\;+\;\gamma w_{ij}+\;\epsilon_{ij} \end{array}$$

Where $\beta_{i}$ are the random coefficients that vary over individuals, and $X_{ij}$ is a vector of alternative-specific variables, γ are fixed coefficients on $w_{ij}$, a vector of alternative-specific variables, and $\epsilon_{ij}$ is a random error term that follows the Gumbel distribution.

The probability of choosing alternative j for respondent i is the standard logistic probability integrated over a density of random parameters,f(β|θ). Stated more explicitly, the choice probabilities of mixed logit model take the following form:


$$\begin{array}{c} P_{ij}=\;\int P_{ij}(\beta_{i})f(\beta )d\beta \end{array}$$

Where


$$\begin{array}{c} P_{ij}\;(\beta_{i})=\;\frac{e^{\beta_{i}X_{ij}\;+\;\gamma w_{ij}}}{\sum_{j=\text{1}}^{\text{5}}e^{\beta_{i}X_{ij}\;+\;\gamma w_{ij}}} \end{array}$$

The integral ∫…dβ indicates that the standard logistic probabilities are averaged over all possible values of β, weighted by the density of each β

Given that price was included as an attribute in the DCE, it was possible to compute MWTP for each characteristic. The MWTP estimates the amount smallholders are willing to pay for a one-unit improvement in a specific attribute of a bull or cow. The MWTP was calculated using the following formula:


$$\begin{array}{c} MWTP=\;-\text{1}\;X\;\left(\frac{\;Coefficient\;of\;the\;attribute}{Coefficient\;of\;price}\right) \end{array}$$

To identify the key driver of smallholder preferences, we calculated the relative importance of the attributes from the MWTP. The relative importance for a specific attribute k was calculated as follows:


$$\begin{array}{c} {Relative\;importance}_{k}=\left(\frac{{MWTP}_{k}}{\sum MWTP\;for\;all\;attributes}\right)*\text{1}\text{0}\text{0} \end{array}$$

The MWTP is suitable for calculating relative importance because it converts utility coefficients into a common monetary scale, allowing direct comparison across attributes with different units. This approach was applied consistently to both the bull and cow models, ensuring that relative importance values are directly comparable.

### Revealed preference

The relationship between observed cattle prices and various influencing factors was analyzed using a hedonic pricing model. This model is based on the premise that the price of a heterogeneous good, such as livestock, is a function of the utility derived from its intrinsic attributes and relevant external factors [33]. In the present study, the hedonic regression provides estimates of the implicit prices (marginal values) for each variable included in the model. The analysis employed the following log-linear functional form of the hedonic price function:


$$\begin{array}{c} \text{ln}\left({Price}_{i}\right)=\beta_{\text{0}}+\;\beta_{\text{1}}({BodyWeight}_{i})+\sum_{k=\text{2}}^{K}\beta_{k}D_{ik}+\varepsilon_{i} \end{array}$$

Where $\text{ln}\left({Price}_{i}\right)$ is the natural logarithm of the observed price for cattle transaction i, $\beta_{\text{0}}$ is the constant term, $\beta_{\text{1}}$ is the coefficient for the continuous variable live body weight, $\beta_{k}$ are the coefficients to be estimated for the k – 1dummy ($D_{ik}$), and $D_{ik}$ represents a set of categorical dummy variables presented in Table 3. For each categorical variable with m level, m−1 dummies are included in the model to avoid perfect multicollinearity. $\varepsilon_{i}$ is the idiosyncratic error term, assumed to be independently and identically distributed.

Homoscedasticity of the error variance is a key assumption for the Ordinary Least Squares (OLS) regression used in the hedonic model. To test this, a Breusch-Pagan-Godfrey test was conducted. The results $F_{\left(\text{2}\text{0},\;\text{1}\text{7}\text{9}\right)}=\text{0}.\text{84},\text{ p}=\text{0}.\text{667 }$indicate that the null hypothesis of homoscedasticity cannot be rejected, confirming that the log-linear functional form of the model is appropriate and that heteroscedasticity is not a concern. Furthermore, the variance inflation factor (VIF) was examined to assess multicollinearity. The VIF values ranged from 1.3 to 3.1, with a mean of 1.8, attesting the absence of severe multicollinearity among the independent variables. Finally, a Ramsey Regression Equation Specification Error Test (RESET) was performed to check for potential model misspecification due to omitted variables. The results $F_{\left(\text{1},\;\text{1}\text{7}\text{8}\right)}=\text{2}.\text{76},\text{ p}=\text{0}.\text{0}.\text{096 }$ demonstrate that the effect of omitted variables is not statistically significant, supporting the adequacy of the specified model.

## Results

### Stated preferences for bull attributes

The marginal utility coefficients for bull attributes, estimated using the mixed logit model, are presented in Table 4. Body size and temperament were specified with random parameters, while all other attributes were modeled as fixed. This specification was guided by their contribution to overall model fit [34]. Attributes that contributed most to overall model fit—based on Log Likelihood changes—were specified as random parameters, while others were modeled as fixed. All evaluated bull attributes had a statistically significant influence on smallholder farmers’ preferences, though the magnitude of their part-worth utilities varied considerably.

**Table 4:** Mixed logit choice model estimates for breeding bull attributes.

Attributes	Coefficient	Std. error	p-values	MWTP*	RI (%)^#^
Fixed parameters					
1. Intercept	base alternative				
2. Intercept	−0.696	0.496	0.161	–	–
3. Intercept	0.038	0.354	0.914	–	–
4. Intercept	−0.318	0.377	0.399	–	–
Draft power strength (strong)	0.350	0.033	0.000	9,346.9	13.0
Disease resistance (tolerant)	0.418	0.068	0.000	11,175.0	15.6
Body coat color (plain)	0.377	0.057	0.000	10,068.8	14.0
Libido (active)	0.269	0.101	0.008	7,185.8	10.0
Purchasing price	−0.000037	0.000011	0.000	–	–
Random parameters					
Body size (large)	0.655	0.062	0.000	17,520.8	24.4
Temperament (docile)	0.613	0.144	0.000	16,384.1	22.9
Standard deviation of random parameters					
Body size (large)	0.001	0.272	–	–	–
Temperament (docile)	0.010	0.358	–	–	–
Log likelihood	−1483.87	–	–	–	–
Number of observations	1536	–	–	–	–
Total	–	–	–	–	100.0

*MWTP – Marginal willingness to pay in Ethiopian Birr (ETB)

#RI – Relative importance in percent

A large body size emerged as the most preferred bull attribute, with a part-worth utility of 0.655. Holding all other attributes constant, smallholders showed a significantly higher preference for large-sized bulls compared to small-sized ones (p < 0.001). Temperament was the second most preferred attribute, with docile bulls favored over aggressive ones (p < 0.001). Economically, body size and temperament were the strongest drivers of preference, together accounting for approximately 47.3% of the total decision-making weight, with individual relative importance scores of 24.4% and 22.9%, respectively. The MWTP estimates further highlight the economic value of these traits. Smallholders were willing to pay an additional 17,520.8 ETB for a large body size and 16,384.1 ETB for a docile temperament relative to their baseline levels. The relatively small standard deviations for these random parameters indicate minimal preference heterogeneity among respondents, suggesting broad consensus on the importance of these attributes.

Disease resistance also significantly influenced smallholders’ preferences, with bulls perceived as “never getting ill” being the most favored (p < 0.001). Coat color was another important trait: bulls with plain coats were strongly preferred over those with mixed colors (p < 0.001). Given the critical role of cattle in agricultural production, smallholders expressed a significantly higher preference for bulls with strong draft power endurance compared to those with weaker strength (p < 0.001). The relative contributions of disease tolerance, plain coat color, and strong draft power to overall utility were similar, with relative importance scores ranging from 13% to 15.6%. Their corresponding MWTP estimates were also comparable, underscoring the economic value farmers attach to these traits.

Active libido was also identified as a desirable attribute, but its relative importance and MWTP were lower compared to other traits, indicating that smallholders placed less emphasis on libido in their breeding decisions. As expected, farmers consistently favored the best set of attributes at the lowest possible price. This preference was confirmed by the significant negative coefficient for the price attribute (p < 0.001), highlighting the sensitivity of smallholders to cost considerations in their choice decisions.

### Stated preferences for cow attributes

The choice preference results for cow attributes, analyzed using a mixed logit model, are presented in Table 5. Milk yield and calf vigor were specified with random parameters, while the remaining attributes were modeled as fixed. The results indicate that both random and fixed parameters significantly influenced smallholders’ preferences for cow attributes.

**Table 5:** Mixed logit choice model estimates for breeding cow attributes.

Attributes	Coefficient	Std. error	p-value	MWTP^*^	RI (%) ^#^
Fixed parameters					
1. Intercept	base alternative				
2. Intercept	0.311	0.282	0.270	–	–
3. Intercept	0.340	0.249	0.171	–	–
4. Intercept	0.075	0.275	0.785	–	–
Body size (large)	0.464	0.061	0.000	6,186.5	19.8
Calving Interval (short)	0.179	0.072	0.013	2,382.7	7.6
Disease resistance (tolerant)	0.222	0.066	0.001	2,967.9	9.5
Temperament (docile)	0.344	0.077	0.000	4,587.9	14.6
Purchasing price	−0.000075	0.000006	0.000	–	–
Random parameters					
Milk yield (high)	0.800	0.071	0.000	10,652.7	34.0
Calf vigor (strong)	0.340	0.058	0.000	4,540.5	14.5
Standard deviation of random parameters					
Milk yield (high)	0.001	0.531	–	–	–
Calf vigor (good)	0.033	0.547	–	–	–
Log likelihood	−1518.392	–	–	–	–
number of observations	1536	–	–	–	–
Total	–	–	–	–	100.0

*MWTP – Marginal willingness to pay in Ethiopian Birr (ETB)

#RPU – Relative importance in percent

The study revealed that milk yield was the most important attribute influencing choice decisions for breeding cows (p < 0.001). This is reflected in its comparatively high relative importance score and the magnitude of its marginal utility. The marginal utility (0.800) for high milk yield was the largest among all attributes included in the model. Furthermore, the MWTP for milk yield was approximately 10,652.7 ETB, demonstrating smallholders’ strong economic valuation of this trait. The small standard deviation estimate (0.001) suggests very low preference heterogeneity for milk yield.

Large body size was the second most preferred cow attribute, with a marginal utility of 0.464. Its relative importance in choice decisions was about 19.8%, while the MWTP for this trait was approximately 6,186.5 ETB. Consistent with the preference observed for bulls, smallholders showed a significantly higher preference for large-sized cows compared to small-sized ones (p < 0.001).

Behavioral characteristics also played a significant role, with docile cows strongly preferred over aggressive ones (p < 0.001). Similarly, calf strength immediately after birth was prioritized, with strong calf vigor favored over weak vigor (p < 0.001). The estimated MWTP and relative importance for calf vigor and temperament were comparable. The standard deviation for calf vigor was about 0.033, indicating negligible preference heterogeneity for this attribute.

A cow's ability to remain healthy when exposed to disease-causing agents also significantly influenced smallholders’ preferences. Overall, cows that were tolerant to disease were preferred over susceptible ones (p < 0.001). Smallholders also showed a significantly higher preference for cows with a short calving interval than for those requiring a longer period between successive births (p < 0.05). However, the relative importance and MWTP for a short calving interval and disease tolerance were lower than anticipated. As expected, the marginal utility of the cow’s purchase price was negative, confirming that smallholders favored desirable attributes at lower prices.

### Odds rations for bull and cow attribute levels

To facilitate interpretation, odds ratios for attribute levels were calculated as the exponentiated coefficients (exp(β)) from the mixed logit model (Table 6). For bulls, the odds ratio of 1.93 indicates that, holding all other attributes constant, the odds of choosing a bull with a large body size were 93% higher than for a small-sized bull. Similarly, the odds of selecting a bull with a docile temperament were 1.85 times higher than for one with an aggressive temperament. For disease tolerance, the odds were 1.56 times higher compared to disease susceptibility.

**Table 6:** Odds ratio with 95% confidence interval for bull and cow attribute preferences.

Attributes and levels	Odds ratio (95% confidence intervals)*	
	Bull	Cow
Body size (Large vs Small)	1.93(1.71, 2.17)	1.59 (1.41, 1.79)
Temperament (Docile vs Aggressive)	1.85(1.39, 2.45)	1.41(1.21, 1.64)
Draft power strength (Strong vs Weak)	1.42 (1.33, 1.51)	–
Disease resistance (tolerant vs Susceptible)	1.52(1.33, 1.74	1.25 (1.10, 1.42)
Coat color (Plain vs Mixed)	1.46(1.30, 1.63)	–
Libido (Active vs Weak)	1.31(1.07, 1.59)	
Milk yield (high vs Low)	–	2.22 (1.93, 2.56)
Calving interval (Short vs Long)	–	1.20 (1.04, 1.38)
Calf vigor (Strong vs Weak)	–	1.41(1.25, 1.58)
Price (High vs Low)	0.96(0.94, 0.98)	0.93 (0.92, 0.94)

*Estimates in the parenthesis are lower and upper confidence intervals of odds ratios

For cows, the odds ratio of 2.22 implies that smallholders were more than twice as likely to choose a cow with high milk yield over one with low yield, assuming all other attributes were equal. This underscores the substantial preference for high-yielding cows. In addition, smallholders had 59% higher odds of selecting cows with a large body size compared to a small size (odds ratio = 1.59).

For both bulls and cows, the odds of selection decreased as price increased. The odds ratios for the high price level were 0.96 for bulls and 0.93 for cows, corresponding to a 4% and 7% reduction in the odds of selection, respectively. These results confirm that while farmers strongly value desirable traits, they remain highly sensitive to purchase price.

### Revealed preferences

The average cattle price during the study period was approximately 38,127 ETB, with a predicted mean live body weight of about 308 ± 95.8 kg (Table 3). Results from the hedonic OLS regression analysis of market transaction data are presented in Table 7. With an adjusted R-squared of 0.92, the model indicates that the included variables collectively explain a high proportion of cattle price variation in the study areas. No statistically significant differences in cattle prices were observed among the three marketplaces (p > 0.05). Similarly, buyers’ educational level had no significant impact on price. In contrast, buyer type was a significant determinant: traders paid a premium approximately 3.7% higher than farmer buyers (p < 0.01). Purchasing purpose also influenced price, with cattle bought for slaughter commanding a premium of about 3.6% compared to those purchased for draft power (p < 0.05).

**Table 7:** Estimates of the hedonic OLS regression model.

Price determinants*	Coefficient	Std. error	p-values
Constant	9.779	0.043	0.000
Market places (Debre Tabor)			
Nefas Mewucha	−0.005	0.015	0.758
Simada	0.004	0.017	0.827
Buyer education level (Elementary school)			
Read and write	0.000	0.018	0.996
Illiterate	0.009	0.014	0.547
Buyer type (Farmer)			
Trader	0.037	0.014	0.010
Purpose of buying (Plowing)			
Breeding	−0.020	0.023	0.367
Resell	−0.002	0.018	0.891
Slaughter	0.036	0.017	0.042
Horn status (Polled)			
Horned	0.016	0.019	0.400
Sex of the cattle (Female)			
Male	0.053	0.020	0.008
Breed of cattle (Crossbreds)			
Local	−0.030	0.018	0.104
Age of cattle (3PPI)			
1PPI	−0.084	0.022	0.000
2PPI	−0.025	0.016	0.115
Full mouth	−0.037	0.018	0.039
Coat color (Mixed)			
Brown	−0.002	0.017	0.907
Black	0.015	0.014	0.287
Body condition (Moderate)			
Good	0.113	0.018	0.000
Live body weight	0.002	0.000	0.000
R-squared 0.93, Adjusted R-squared = 0.92, F-statistics = 131.2, p-value = 0.000			

* Variables within the parentheses are the base category

Among the various biological characteristics investigated, sex, age, body condition, and live body weight were the main sources of cattle price variation in the study areas (Table 7). In contrast, breed type, horn status (presence/absence), and coat color did not have a significant influence on price (p > 0.05).

Regarding the effect of sex, male cattle were significantly more expensive than females, with a price premium of approximately 5.1% (p < 0.01). Age was estimated based on dental eruption patterns. It was observed that animals in the younger age category (1PPI) and those older than five years (full mouth) were marketed at lower prices compared to those in the 3PPI category (p < 0.05). In other words, age and price exhibited a non-linear relationship: price increased up to a certain age but declined as animals became older. There was no significant price difference between animals in the 2PPI and 3PPI age categories (p > 0.05).

The present study revealed that cattle body condition is a crucial price determinant in the local market. The market price for cattle in good body condition was about 11.3% higher than for those in moderate body condition. This result implies that buyers place heavy reliance on the body condition status of an animal when making purchase decisions. Live body weight also significantly influenced price. Holding all other factors constant, the price increased by approximately 0.2% for every additional kilogram of live weight (Table 7). Fig 2 also demonstrates that live body weight and the logarithm of price have a strong linear relationship, with heavier animals commanding a higher premium.

**Fig 2:**
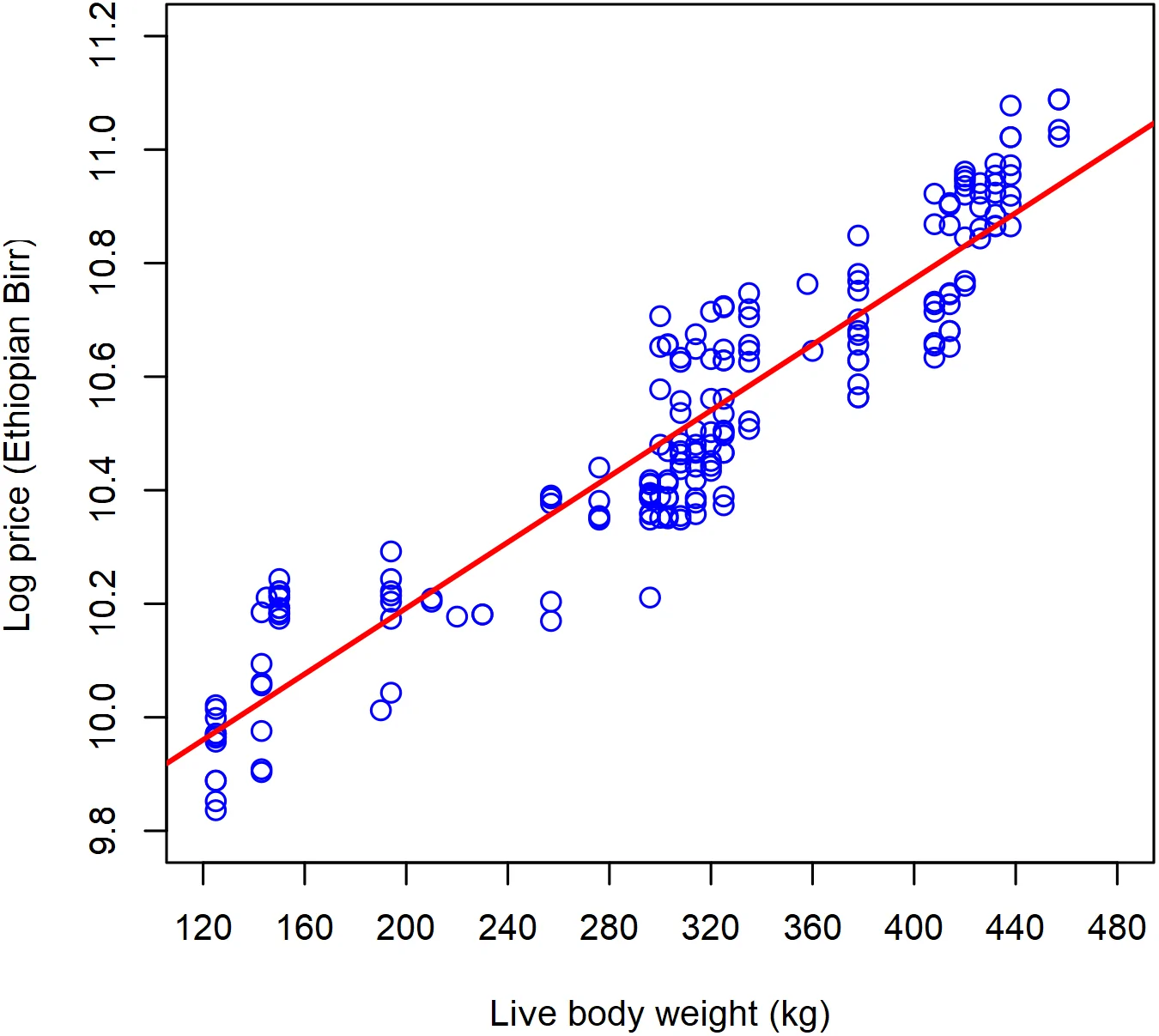
Relationship between live body weight and the natural logarithm of price.

## Discussion

Harnessing indigenous cattle genetic resources as a pathway to improve the livelihoods of smallholders in developing countries, such as Ethiopia, largely depends on efforts to enhance their overall productivity [2,35]. As an initial step toward designing a conservation-based breeding program, this study investigated smallholders’ preferences for cattle traits and assessed the internal and external factors influencing cattle prices in northwest Ethiopia. The results of the choice modeling revealed that multiple attributes significantly influenced smallholders’ preferences for indigenous cattle. The hedonic regression analysis also identified that both cattle biological characteristics and external market factors, such as buyer type and purchase purpose, were significant determinants of price variation in the local market.

### Synthesis of stated and revealed preferences for trait prioritization

This study integrated DCE and hedonic pricing approaches to identify valuable cattle traits from both producer preference and market perspectives. The DCE revealed that large body size was the first and second most preferred trait for bulls and cows, respectively. This strong preference for body size was strongly corroborated by the hedonic pricing analysis, in which superior body condition and greater live body weight exerted a significant positive influence on cattle market prices. This convergence between stated and revealed preferences validates smallholders’ trait prioritization and provides robust evidence for including body size as a primary breeding objective. Scarpa et al. [36], employing a similar mixed-methods approach with indigenous cattle in Kenya, found that larger body size significantly influenced both choice preference and market price. Similarly, Woldu et al. [16] reported convergence between DCE and hedonic model results with respect to body size in Ethiopian indigenous goats. These consistent findings across species and geographic contexts suggest that body size is a robustly valued trait in smallholder livestock systems.

Milk yield was the most preferred cow trait identified in the DCE. However, unlike body condition and live body weight—which are visually apparent at the market—milk yield is not directly observable at the point of transaction and as a result, could not be incorporated into the hedonic pricing model. This absence reflects a measurement limitation rather than a lack of market valuation. The high MWTP for milk in the DCE indicates that farmers value this trait strongly, but it cannot be priced through observable characteristics at the point of sale. This finding underscores the value of stated preference methods in capturing traits that are important to farmers but cannot be measured through market transactions. Therefore, while market data validates the economic importance of body size, the DCE results emphasize that breeding objectives must also prioritize milk production to align with smallholders’ livelihood strategies. The consistent emphasis on body size and milk yield aligns with previous reports indicating that indigenous cattle are crucial sources of food (milk and meat) and cash income at the smallholder level [2,3,37].

Apart from milk yield, traits such as docile temperament, disease tolerance, strong draft power, active libido, strong calf vigor, and short calving interval are not directly observable at the point of sale and were therefore not captured in the hedonic model. Their absence from the hedonic model does not imply that they are undervalued by the market; rather, it reflects the inherent difficulty of pricing such traits through observable transaction characteristics. In contrast, the hedonic model revealed market structure effects invisible to the DCE—for example, that traders pay higher prices and that slaughter animals command a premium. These divergences suggest that breeding for higher-value slaughter markets could increase profits but may conflict with smallholders’ draft power needs.

Beyond observability constraints, the absence of record-based livestock transactions in Ethiopia contributes to significant information asymmetries in the market, particularly regarding reproductive and adaptive performance. Without formal records or transaction histories, buyers cannot verify an animal's health status, reproductive history, or productivity potential at the point of sale. This information asymmetry—not a lack of buyer interest—explains why traits such as disease resistance, reproductive efficiency, and temperament are not reflected in market prices. Taken together, these findings clearly highlight the value of integrating stated and revealed preference methods to identify robust breeding objective traits.

Notwithstanding the merits of combining both strategies, certain methodological warrant attention. First, while the DCE and hedonic pricing analyses targeted the same population of smallholder cattle keepers in northwest Ethiopia, the actual samples differed in composition and context: the DCE was administered to randomly selected farmers in their villages, whereas the hedonic model relied on transaction data collected at livestock markets. Consequently, the market sample included traders and other buyers —such as farmers acting as buyers or consumers—who were not fully represented in the village-based DCE sample. This difference may partly explain some of the observed divergence between the two preference measures. As such, future research should consider integrated sampling designs—collecting stated preferences from farmers and subsequently tracking their actual market transactions—to minimize such divergence. Second, body live was estimated using Schaeffer's formula—a widely used method in field-based livestock studies [38–40]. Despite its popularity, this approach may introduce measurement error and lead to attenuation bias [41]. Our estimate of the price premium is therefore likely conservative, though the coefficient remains highly significant (p < 0.001).

### Implications for designing a conservation-based breeding program

The convergence and divergence between stated and revealed preferences observed in this study provide a robust empirical foundation for defining breeding objectives that balance smallholder priorities with market realities. The strong alignment between farmers’ preferences for large-framed cattle and market premiums for superior body condition and live body weight suggests that growth rate and mature size should be primary breeding objectives. Genetic improvement of these traits would simultaneously satisfy breeder preferences and enhance market value, thereby creating a stronger economic incentive for sustainable resource conservation and utilization.

However, the high stated preference for milk yield—despite its invisibility in local market transactions—highlights the need for breeding objectives that extend beyond what prices alone reveal. Milk production is vital to smallholders’ household consumption, nutrition, and daily income generation [42,43], yet it cannot be captured in market prices because it is not directly observable at the point of animal sale. Thus, adopting a dual-purpose breeding objective that incorporates both body size and milk yield is vital for a conservation-based breeding program within smallholder systems. In addition, breeding objectives should weigh these traits based on their relative contributions to smallholder livelihoods, rather than solely on their market premiums.

Furthermore, the finding that buyer type and purchase purpose significantly influence prices suggests that market segmentation offers opportunities for niche breeding strategies. For instance, cattle destined for slaughter commanded higher prices, indicating that selecting for traits valued in the beef market—such as conformation and finish—could target this premium segment. However, such an approach must account for the continued need for draft power and milk production within smallholder systems. Beyond the traits discussed above, the DCE revealed that smallholders value several other invisible traits—including docile temperament, disease tolerance, strong draft power, active libido, strong calf vigor, and a short calving interval—which are not captured in market prices due to observability constraints at the point of sale.. While incorporating all of these into a breeding objective may be challenging, breeding programs should, at a minimum, avoid selecting against them. Care must be taken to monitor potential genetic correlations between visible and invisible traits to prevent unintended declines in these livelihood-critical attributes.

The relatively small standard deviations indicate broad consensus among farmers on the importance of body size, temperament, and milk yield. While the standard deviation for calf vigor was somewhat larger, it remains modest. These findings suggest that a single breeding objective emphasizing these traits would serve the majority of smallholder farmers. This is particularly important in the context of smallholder breeding programs, where collective action and a large breeding population are required, making individualized breeding objectives impractical.

## Conclusion

Integrating stated and revealed preference approaches has enabled the identification of priority traits that can inform breeding objective definition by reconciling farmers’ preferences with market incentives. The observed convergence suggests that body size should be considered a primary candidate breeding objective, while divergence on milk yield and other non-observable traits (e.g., docile temperament, disease tolerance, libido, calving interval, and calf vigor) reveals that these traits are not captured in market prices due to observability constraints and information asymmetry. Combining both methods is therefore valuable—hedonic models alone miss invisible traits, while DCE alone misses real market structures such as price premiums for traders and slaughter animals. These findings provide empirical inputs for breeding objective definition and suggest that breeding programs consider multi-trait objectives that balance market competitiveness with household livelihood needs, incorporating both visible and invisible traits. Developing a formal selection index for these traits will require additional steps—namely, estimating genetic parameters and economic weights—which represent important directions for future research. This integrated approach can help ensure that conservation-based breeding programs enhance, rather than compromise, the full range of smallholder livelihood strategies.

## Supporting information

S1 File Choice preference raw data. It is an Excel file (XLSX) containing raw data for bull and cow choice preference analysis.
(XLSX)

S2 File Revealed preference raw data. It is an Excel file (XLSX) containing raw market data for hedonic pricing analysis.
(XLSX)
