# Supplementary material for: Combining stated and revealed preferences to define smallholder cattle breeding objectives in northwest Ethiopia: Evidence for breeding program design
Source: PLoS One. 2026 Oct 7;21(10):e0359944. doi: 10.1371/journal.pone.0359944 (PMC13645039; doi:10.1371/journal.pone.0359944)
Supplement: Supplementary file 1 [file pone.0359944.s003.pdf]

Response to Reviewers

Manuscript ID: PONE-D-26-27814

Manuscript title: Combining stated and revealed preferences to define smallholder cattle breeding objectives in northwest Ethiopia: Evidence for breeding program design

Dear Editor and Reviewers,
We greatly appreciate your comments and suggestions on our manuscript. We have carefully addressed your valuable insights in our revisions and hope they now meet your expectations. Below, we provide detailed responses to each point.

I. Comments from the Editor

Journal Requirements:

When submitting your revision, we need you to address these additional requirements.

1. Please ensure that your manuscript meets PLOS ONE's style requirements, including those for file naming. The PLOS ONE style templates can be found at

https://journals.plos.org/plosone/s/file?id=wjVg/PLOSOne_formatting_sample_main_body.pdf andhttps://journals.plos.org/plosone/s/file?id=ba62/PLOSOne_formatting_sample_title_authors_affiliations.pdf

Authors' Response:
We thank the Editor for reminding us of this important issue. The authors have carefully prepared the manuscript in strict accordance with PLOS ONE's guidelines and have made every effort to ensure full compliance with the journal's style requirements.

2. If the reviewer comments include a recommendation to cite specific previously published works, please review and evaluate these publications to determine whether they are relevant and should be cited. There is no requirement to cite these works unless the editor has indicated otherwise.

Please review your reference list to ensure that it is complete and correct. If you have cited papers that have been retracted, please include the rationale for doing so in the manuscript text, or remove these references and replace them with relevant current references. Any changes to the reference list should be mentioned in the rebuttal letter that accompanies your revised manuscript. If you need to cite a retracted article, indicate the article's retracted status in the References list and also include a citation and full reference for the retraction notice.

Authors' Response:

We thank the Editor for this important reminder. The reviewers did not recommend citing any specific previously published works. However, we have added several references while addressing the reviewers' comments and questions. The full reference list has been reviewed for completeness and correctness, and all references are included at the end of the manuscript. We confirm that no

retracted articles have been cited in our manuscript. All changes to the reference list are reflected in the revised manuscript.

Additional Editor Comments:

In light of the reviews received, I kindly ask the authors to incorporate the suggestions made by the reviewers. To facilitate the re-evaluation process, please ensure that all changes and adjustments made to the manuscript are clearly highlighted in a different color. Once the revised version is submitted, we will proceed with the editorial process for the publication of the work.

[Note: HTML markup is below. Please do not edit.]

Authors' response:

We thank the Editor for this clarification. As required, all changes and adjustments made to the manuscript have been clearly highlighted in a different color using the track changes function in Microsoft Word. In accordance with the journal's requirement, we submitted two files:

Manuscript with track changes – showing all modifications in a different font color (red/blue) for easy identification.

Clean revised manuscript – without track changes, representing the final version for publication.

II. Comments and questions from Reviewer #1

Reviewer #1: Please find below the primary evaluation, constructive, and simple feedback for the manuscript referenced (PONE-D-26-27814), entitled 'Integrating Expressed and Inferred Preferences for Setting Livestock Breeding Goals among Smallholder Farmers in Northwest Ethiopia: Guidelines for Designing Breeding Programs'. The comments provided below address the most significant methodological and interpretative issues essential for enhancing the rigor of the manuscript:

Authors' Response:

We thank the reviewer for their careful reading of our manuscript. We note that the reviewer refers to our manuscript as "Integrating Expressed and Inferred Preferences for Setting Livestock Breeding Goals among Smallholder Farmers in Northwest Ethiopia: Guidelines for Designing Breeding Programs."

We would respectfully clarify that our original submitted title is:

"Combining stated and revealed preferences to define smallholder cattle breeding objectives in northwest Ethiopia: Evidence for breeding program design"

We believe this title more accurately reflects the scope and contribution of our study for the following reasons:

"Guidelines" implies prescriptive protocols, whereas our study provides empirical evidence and inputs to inform breeding program design. We have therefore retained "Evidence for breeding program design."

"Livestock" is too general, as our study specifically focuses on cattle.

"Stated preference" and "revealed preference" are the established terms in the DCE and hedonic pricing literature, respectively, and precisely describe our methodological approach.

We hope this clarifies our title and appreciate the reviewer's engagement with our work.

1) Measurement Error & Potential Attenuation Bias (Reviewer #1)

The Problem: Body weight was not measured using actual scales at the market; instead, it was estimated using Schaeffer's formula based on heart girth and body length measurements taken with a tape. Introducing an estimated variable as a continuous regressor in an Ordinary Least Squares (OLS) model introduces measurement error. This typically causes attenuation bias (biasing the coefficient toward zero), which may explain the very small coefficient observed for body weight.

Proposed solution & correction: The authors must explicitly acknowledge this methodological limitation in the discussion section. They should also perform a sensitivity analysis or discuss how this measurement error might have deflated the perceived economic weight of the animal's size in the hedonic model.

Authors' Response:
We thank the reviewer for this important methodological observation. We have acknowledged this limitation in the Discussion section. However, we would also like to demonstrate that the economic significance of live body weight remains meaningful despite potential attenuation bias. Our hedonic model yielded a coefficient of 0.002 ($p < 0.001$) for live body weight, corresponding to a 0.2% price increase per additional kilogram. With a mean cattle price of 38,127.2 ETB (Table 3), this equates to approximately 76 ETB per kg. For example, a 50 kg increase in live body weight—well within the observed range—corresponds to nearly 3,800 ETB, or a 10% price premium. In the context of smallholder livelihoods in rural Ethiopia, this represents a substantial economic return.

Therefore, while we acknowledge that measurement error may introduce some attenuation bias, the economic significance of live body weight remains clearly evident even under a conservative estimate. We have added a concise limitation paragraph to describe this limitation in the discussion (page24, lines 461 – 465)

2) Confounding Effect Due to Sample Incompatibility (Reviewer #1)

The Problem: The manuscript attempts to directly compare "stated preferences" (DCE) with "revealed preferences" (Hedonic Pricing). However, the two samples represent completely different populations. The DCE was conducted exclusively with smallholder farmers in their villages, while the market transactions included traders, butchers, and urban consumers alongside farmers. Comparing these two datasets introduces a confounding factor: the difference in preferences might not be due to "market opacity" as the authors claim, but simply due to the differing economic objectives of the buyers (e.g., traders looking for immediate resale value vs. farmers looking for long-term breeding traits).

The Solution: The authors need to stratify or filter the market dataset, if possible, to isolate transactions made by farmers buying for breeding purposes. If the data does not allow for this distinction, this major population mismatch must be thoroughly discussed as a primary caveat that limits the direct comparability of the two models.

Authors' Response:
We thank the reviewer for this observation. We would like to clarify the methodological rationale underlying our comparative approach and note that this limitation was also already acknowledged in our original manuscript.

- Regarding *"...the two samples represent completely different populations."* we would respectfully clarify that the two samples are not "completely different populations" but rather substantially overlapping populations drawn from the same study area. Both the DCE and hedonic data were collected from the same three districts in northwest Ethiopia. The DCE sample consisted entirely of smallholder farmers (100%), while the hedonic market sample consisted of 55% farmers and 45% traders. This means that the majority of market transactions involved the same population (farmers) surveyed in the DCE.
- **Separate analysis eliminates confounding risk**: Importantly, the reviewer's concern about confounding effects would be serious had we merged the two datasets and analyzed them together. However, as stated in the "Study approach" subsection, the datasets were analyzed separately and independently:

  - The DCE was analyzed using a mixed logit model to estimate farmers' stated preferences.
  - The hedonic model was analyzed using a log linear OLS regression to estimate market-determined trait values.
  - The results were subsequently compared to identify convergence and divergence.

This approach avoids the methodological pitfalls of merging incompatible datasets. Each model is internally valid, and the comparison is **interpretive rather than statistical**—thus minimizing confounding risk.

- **Controls for sample composition:** Our hedonic model explicitly controlled for buyer type and purchase purpose (Table 7). Traders paid 3.7% more than farmers ($p = 0.010$), and animals for slaughter commanded a 3.6% premium over those for ploughing ($p = 0.042$). Despite these controls, our core findings remained robust.

- **Complementary, not contradictory, samples:** The two samples capture different facets of the same population: The DCE captures stated preferences of farmers in their villages. The hedonic model captures revealed preferences from market transactions, which include farmer-buyers (55%) and traders (45%).
- **Breeding objectives must address both demand and supply**: A breeding program must serve both farmer preferences (what farmers want in their herds) and market demand (what buyers are willing to pay for). Both perspectives are essential for designing a breeding program that is biologically sound and economically viable.
- **Limitation already acknowledged:** We note that this limitation was already acknowledged in the original manuscript's Discussion section as follows:

"Notwithstanding the merits of combining both strategies, certain methodological warrant attention. First, while the DCE and hedonic pricing analyses targeted the same population of smallholder cattle keepers in northwest Ethiopia, the actual samples differed in composition and context: the DCE was administered to randomly selected farmers in their villages, whereas the hedonic model relied on transaction data collected at livestock markets. Consequently, the market sample included traders and other buyers —such as farmers acting as buyers or consumers—who were not fully represented in the village-based DCE sample. This difference may partly explain some of the observed divergence between the two preference measures. As such, future research should consider integrated sampling designs—collecting stated preferences from farmers and subsequently tracking their actual market transactions—to minimize such divergence…"

We have further refined this discussion in the revised manuscript taking into account the previous comments in live body weight estimation.

3) Misinterpretation of Log-Linear Regression Coefficients (Reviewer #1)

The Problem: In the Hedonic Pricing model (T. 7), the coefficient for body weight is reported as 0.002. In the text, the authors interpret this as a 0.02% increase in market price per additional kilogram of body weight. This is a mathematical error. In a log-linear model (\(\ln(Y) = \beta_0 + \beta_1 X\)), the percentage change in Y for a unit change in X is calculated as \((e^{\beta_1} - 1) \times 100\), which for small coefficients is approximately $\beta_1 \times 100$. Therefore, a coefficient of 0.002 corresponds to a 0.2% increase, not 0.02%.

Proposed solution & correction: The authors must correct this mathematical interpretation throughout the manuscript and re-align their discussion to reflect the actual magnitude of the effect.

Authors' Response:

We thank the reviewer for pointing out this error. We noted that while interpreting live body weight coefficient, we mistakenly wrote 0.02% in the text, which should be 0.2%. We made the correction in the text and now the text reads:

"… Holding all other factors constant, the price increased by approximately 0.2% for every additional kilogram of live body weight…"

4) Methodological Implausibility of Zero Opt-out Choices (Reviewer #1)

The Problem: The authors state that out of the 384 farmers surveyed, none chose the "Opt-out" (status quo / none of the choices) option across all choice sets. In Discrete Choice Experiments (DCE), a zero frequency for the opt-out alternative is highly unusual and statistically improbable. This raises concerns about potential enumerator bias (e.g., field interviewers rushing respondents or discouraging them from choosing the opt-out) or a fundamental misunderstanding of the baseline option by the respondents.

Request for Clarification: The authors must provide a rigorous methodological justification for this outcome. They need to explain how the choice tasks were explained to the smallholders, how interviewers were trained to handle the opt-out option, and discuss whether this lack of variation forces any bias into the Mixed Logit model estimates.

Authors' response:
We thank the reviewer for this excellent and methodologically rigorous comment. A zero or near-zero selection rate for the opt-out alternative is indeed uncommon in standard consumer-market DCEs. However, in the specific context of defining livestock breeding objectives within smallholder farming systems, this outcome is both behaviorally rational and methodologically documented. For instance, Abebe et al. (2020) reported a frequency of opt-out below 0.5% in a sample of 370 households following a DCE study on sheep, while Kassie et al. (2009) reported a 1.4% opt-out frequency from a sample of 195 for valuing traits of indigenous cows in central Ethiopia. These negligible opt-out choices clearly demonstrate how context dictates choice behavior. We address the reviewer's specific points as follows:

- **Methodological & Contextual Justification:**
  - **The framing of the opt-out:** In our DCE, the opt-out alternative was explicitly framed to farmers as: "No genetic improvement or trait modification is needed for your herd relative to the presented options." Since the objective of the study was to identify breeding preferences (traits to be genetically improved), framing the opt-out in this manner was highly appropriate.
  - **The reality of smallholder mixed crop-livestock production systems:** Cattle are a critical asset for smallholder farmers, serving as a household food supply, income source, and draught power for crop production. Given that indigenous cattle in Ethiopia are the result of natural selection, they are well-adapted to low-input systems, though their average productivity remains generally low. For a smallholder whose livelihood depends heavily on these animals, the premise that "no improvement is needed" is fundamentally counter-intuitive. Because they actively desire and need better-performing animals to secure food and income, choosing the status quo (the opt-out) was viewed as an unrealistic and non-viable strategy.

- **Minimized complexity in design**: As described in the methodology, we used a fractional factorial design blocked into four parts to minimize cognitive demand, keeping in mind the lower literacy levels typical of smallholder communities. The design was limited to seven attributes, with all having two levels except price, which had three. This streamlined setup ensured that respondents did not experience cognitive fatigue or confusion—common drivers of uninformative opt-out choices in complex DCEs.

➢ **Field protocol and enumerator training (addressing potential bias)**

While it is impossible to eliminate all potential bias in field research, we implemented a rigid quality-control protocol during fieldwork to minimize biases arising from miscommunication:

- **Enumerator expertise:** All enumerators held a BSc degree in Animal Science. They underwent an intensive training program covering respondent interaction, the basics of valuing livestock traits using DCEs, and the attribute levels presented on each choice card. This included mock interviews specifically focused on neutrality. Enumerators were strictly instructed to read all five alternatives—including the opt-out—with equal emphasis and without prompting or rushing the respondent.
- **Visual support:** We utilized tailored visual aids for the majority of the trait levels (see Figure 1 in the manuscript). Before commencing the choice tasks, enumerators briefed the participants on the study's purpose and consistently used these visual cards to clearly explain every alternative.
- **Strict supervision:** Field supervisors conducted random spot-checks during interviews to verify that all alternatives, including the opt-out, were being presented transparently. The zero-frequency trend was uniform across all enumerators, ruling out isolated interviewer bias or systematic rushing.

➢ **Econometric implications & potential model bias**

- **Model convergence & mathematical necessity:** The reviewer is correct that a complete lack of variation in a choice category prevents standard parameter estimation. Had we included the opt-out alternative with an all-zero outcome vector in the final Mixed Logit specification, the model would fail to converge because the Alternative-Specific Constant (ASC) for the opt-out would approach negative infinity. Mathematically, it must be excluded from the estimation matrix, and we did so.
- **Preserving the unforced choice nature:** Excluding the unselected opt-out from the statistical analysis does not retrospectively transform the experiment into a forced-choice design. The behavioral choices were freely made by the respondents in the field, as the opt-out option was physically present on the choice cards.
- **Relaxing the IIA assumption via Mixed Logit:** A common concern when altering choice sets in a standard Multinomial Logit (MNL) model is the violation of the Independence from Irrelevant Alternatives (IIA) assumption. However, we utilized a Mixed Logit (MXL) model, which natively relaxes the IIA restriction by allowing parameters to vary randomly across individuals.
- **Captured in the error and heterogeneity terms:** Because the MXL model accommodates unobserved taste heterogeneity, any residual behavioral variance or latent effects related to the baseline/opt-out option—which respondents deliberately bypassed—

are appropriately absorbed into the model's random parameter distributions and idiosyncratic error terms, rather than biasing the core trait preference estimates.

In addition the above explanation, we included the following text in the revised manuscript (lines 174 to 184) to further clarify the issue:

"*The zero selection rate for the opt-out alternative, while uncommon in consumer-market choice experiments, is both behaviorally rational and methodologically documented within smallholder farming systems. For instance, Abebe et al. [12] reported an opt-out frequency below 0.5% for sheep traits, while Kassie et al. [28] observed a 1.4% opt-out rate for indigenous cow traits in Ethiopia. Because the explicit goal of the choice task was to define the breeding objective, the opt-out alternative was framed to farmers as: "no genetic improvement or trait modification is needed for your herd relative to the presented options." Given the subsistence constraints of the crop-livestock production system, selecting the status quo was viewed as unviable by the respondents, illustrating how local context dictates choice behavior. Consequently, due to this complete lack of statistical variation, the opt-out alternative was excluded from the final econometric estimation matrix to ensure model convergence."*

III. Comments and questions from Reviewer #2

Reviewer #2: Major Comments

1) Clarification of the relationship between stated and revealed preferences (Reviewer #2)

The two approaches are presented as a 'combined' methodology, but the samples and data sources used in the two analyses were different, and the analyses were performed separately. The authors must explain that the study is a comparative and complementary integration of the evidence, but not a statistical integration of the two sets of evidence. Standard terms should be used in the manuscript throughout.

Authors' Response:

We thank the reviewer for this important clarification. We fully agree that the manuscript should clearly state that the two datasets were analyzed separately and not statistically integrated. We would like to note that this was already stated in the manuscript's "Study approach" subsection as:

"... Although both methods focused on the same target population, the specific respondents and data sources differed. Consequently, the datasets were analyzed separately rather than merged, and their results were subsequently compared to provide complementary insights."

However, we acknowledge that this statement could be more explicit. We have therefore revised the "Study approach" subsection of the manuscript to further emphasize this as follows:

"Study approach

This study employed both DCE and hedonic pricing methods to examine smallholder preferences for indigenous cattle traits. The target population for both methods was smallholder farmers in

the selected districts. Choice data for the DCE were collected through a cross-sectional survey administered to randomly selected farmers, while transaction data for the hedonic pricing method were obtained by observing cattle sales in the major markets of the study districts. Although both methods focused on the same target population, the specific respondents and data sources differed. Consequently, the datasets were analyzed separately and independently rather than merged, and their results were subsequently compared and integrated to provide complementary insights. ***This study therefore represents a comparative and complementary integration of evidence, not a statistical integration of the two datasets."***

Regarding clarification on "combining" vs. "comparing" in the title: We respectfully retain the term "combining" rather than "comparing" for the following reasons:

- **Purpose of the study:** Our goal was not to test which method performs better, but to integrate insights from both methods to define a balanced breeding objective that reflects both farmer preferences (DCE) and market demand (hedonic pricing).
- **Complementary strengths:** As described in the Introduction, each method has distinct strengths and weaknesses. Combining them allows us to capitalize on their respective strengths and mitigate their individual weaknesses.
- **Reader expectations:** If we used "comparing" in the title, readers would expect a verdict on which method is superior. Instead, our conclusion is that employing both methods together provides a more complete basis for defining breeding objectives.

We have also audited the manuscript to ensure consistent use of terminology. We are grateful to the reviewer for prompting this clarification, which has improved the precision of our manuscript.

2) Interpretation of non-observable traits in the hedonic model (Reviewer #2)

Where traits like milk yield, disease resistance, temperament, libido, and reproductive traits are missing from the hedonic model, the manuscript uses that as grounds to conclude that these traits are "undervalued by the market." The above interpretation may be extreme inasmuch as those traits were not directly observed or reliably documented at the point of sale. The authors need to distinguish between lack of market valuation and the inability to describe the trait through transaction prices that are observed.

Authors' Response:

We thank the reviewer for this extremely important and insightful observation. We fully agree that our original interpretation conflated two distinct concepts: (1) lack of market valuation and (2) the inability to measure a trait through observed transaction prices. We have revised the Discussion section accordingly.

Specific revisions made:

Replaced *"the meat-focused market undervalues"* with: *"This absence reflects a measurement limitation rather than a lack of market valuation."*

Replaced *"invisible to the market"* with: *"not directly observable at the point of sale"* and added: "*Their absence from the hedonic model does not imply that they are undervalued by the market; rather, it reflects the inherent difficulty of pricing such traits through observable transaction characteristics."*

Added explicit statement: *"This information asymmetry—not a lack of buyer interest—explains why traits such as disease resistance, reproductive efficiency, and temperament are not reflected in market prices."*

Strengthened the role of DCE: *"This finding underscores the value of stated preference methods in capturing traits that are important to farmers but cannot be measured through market transactions."*

We are grateful to the reviewer for prompting this important clarification, which has significantly improved the accuracy and nuance of our interpretation.

3) The hedonic analysis sample size and model complexity (Reviewer #2)

The hedonic model includes a relatively large number of levels of categorical variables and explanatory variables but is based on just 200 transactions. The authors should present a more compelling argument regarding the appropriateness of the sample size in the context of the number of parameters estimated and the stability and generalizability of the estimates.

Authors' Response:

We thank the reviewer for this important observation. We would like to clarify that the appropriateness of our sample size and the stability of our model have been addressed in the manuscript.

- **Sample size justification (revised methods section):** There is no single dedicated formula for calculating sample size specifically for hedonic pricing models. Instead, researchers rely on standard multivariable OLS regression guidelines. Harrell (2015), in his authoritative book on regression modeling strategies, discussed a nuanced set of rules of thumb regarding the relationship between sample size (m) and the number of predictors (p):
    - Liberal rule: $p < m/10$, $200/10 = 20$
    - Good average rule: $p < m/15 = 200/15 = 13.33$
    - Conservative rule: $p < m/20 = 200/20 = 10$

  With m = 200 and p = 11 predictors, our sample size of 200 satisfies both the liberal and "good average" rules of thumb, while it falls slightly short of the conservative criterion. This implies that our sample size was reasonably adequate to estimate the effect of 11 predictors without overfitting the model. Achieving a higher sample size is often difficult in field-based livestock market studies, especially in the study area where standardized livestock marketing is absent.

We have included the following explanation in the revised Methods section (lines 198 to 205):

"*The market transaction dataset includes 10 categorical and one continuous variables, with 11 predictors in total. According to Harrell [28], the ratio of sample size (m) to number of predictors (p) should satisfy p < m/10 (liberal), p < m/15 (good average), or p < m/20 (conservative). With m = 200 and p = 11, our study satisfies both the liberal rule (11 < 20) and the good average rule (11 < 13.33), implying the sample size was reasonably adequate to estimate the effects of 11 predictors without introducing overfitting. Achieving a higher sample size is often difficult in field-based livestock market studies, especially in the study area where standardized livestock marketing is absent.*"

- **Model stability and diagnostics (existing Methods section):** The manuscript also reports comprehensive diagnostic tests confirming model stability:

Multicollinearity was assessed using VIF (range 1.3–3.1; mean 1.8). The Breusch-Pagan-Godfrey test confirmed homoscedasticity ($p = 0.667$), and the Ramsey RESET test indicated no significant misspecification ($p = 0.096$). Adjusted $R^2 = 0.92$ and F-statistic = 131.2 ($p < 0.001$) indicate excellent model fit.

4) The use of forecasted as opposed to measured body weight (Reviewer #2)

Heart girth and body length were used to estimate body weight by applying Schaeffer's formula. The authors should explain why it is appropriate to use predicted body weight instead of direct weighing and should explain the possible measurement error that may arise from the prediction equation and the influence it may have on the hedonic price estimates.

Authors' Response:

We thank the reviewer for this important observation. We acknowledge that body weight was estimated using Schaeffer's formula rather than measured directly with scales, and we appreciate the opportunity to explain why this approach was necessary and how it may affect our estimates.

- **Why Schaeffer's formula was used**: We used Schaeffer's formula because direct weighing of cattle at livestock markets in rural Ethiopia presents significant practical challenges due to the absence of weighing scales. Livestock markets in the study area are not equipped with cattle weighing scales. This is consistent with the broader context of livestock marketing in Ethiopia, where standardized marketing infrastructure is largely absent. Schaeffer's formula is a widely recognized and validated method in livestock research where direct weighing is not feasible (e.g., Wangchuk et al., 2017; Los et al., 2022; Ganapathi et al., 2025).
- **Measurement error and attenuation bias:** We acknowledge that using predicted body weight introduces measurement error. In an Ordinary Least Squares (OLS) framework, classical measurement error in a continuous regressor typically leads to attenuation bias, where the estimated coefficient is biased toward zero (Wooldridge, 2010). Under this assumption, our estimate of the price premium for body weight (0.002, or 0.2% per kg) is likely conservative. While the direction of bias depends on the nature of the measurement error, the

coefficient for body weight remains highly significant ($p < 0.001$), reinforcing the importance of body size as a market-valued trait. We have clearly described this limitation in the Discussion section (lines 461 to 465 of the revised manuscript).

- **Influence on hedonic price estimates**: Under the classical measurement error assumption, the direction of bias is toward zero, which means our findings on body weight represent a lower bound of its true economic value. Other coefficients in the model are less likely to be affected, as they are based on directly observed categorical variables. The overall model fit (adjusted R^2 = 0.92) and diagnostic test results suggest that the model remains robust despite this limitation.

We are grateful to the reviewer for prompting this clarification.

5) Very high explanatory power of the hedonic model (Reviewer #2)

The adjusted R^2 value reported is very high for a cross sectional livestock market model. This is not necessarily a sign of a problem, but the authors should discuss this further, discuss possible overfitting, and indicate whether other model specifications gave similar results.

Authors' Response:

We thank the reviewer for this thoughtful observation. We appreciate the opportunity to clarify the high explanatory power of our hedonic model.

- **Why high explanatory power is plausible in this context:** Cattle are relatively homogeneous assets traded based on observable characteristics—body condition, live weight, sex, age, breed, and coat color. In rural Ethiopian livestock markets, where formal grading and performance records are absent, negotiations are based on these directly observable traits. Our model captures these variables comprehensively, explaining the high adjusted R^2.
- **Overfitting concerns**: The diagnostic tests relevant to overfitting are already reported in the Methods and Results sections:
    - Minimal difference between R^2 and adjusted R^2: The R^2 is 0.93 and the adjusted R^2 is 0.92, a difference of only 0.01. Since adjusted R^2 penalizes for the number of predictors, this minimal drop suggests no severe overfitting.
    - Reasonably adequate sample size relative to predictors: 200 observations / 11 predictors = 18:1 ratio, exceeding established thresholds (Harrell, 2015).
    - Low multicollinearity: VIF values range from 1.3 to 3.1 (mean 1.8), well below the threshold of 5.
    - No significant misspecification: The Ramsey RESET test ($p = 0.096$) indicates no significant omitted variable bias.
- **Alternative model specifications**: To assess robustness, we estimated the model using an alternative functional form (linear-linear specification). The results were as follows:

Model Specification	R^2	Adjusted R^2	Breusch-Pagan-Godfrey (p-value)	Verdict
Log-linear (primary model)	0.93	0.92	0.667	Homoscedasticity confirmed

Linear-linear (alternative)	0.90	0.89	0.060 (F-test); 0.008 (Scaled SS)	Evidence of heteroscedasticity

The linear-linear specification yielded a similarly high adjusted R^2 (0.89), confirming the robustness of the model's explanatory power. However, this functional form exhibited evidence of heteroscedasticity (Breusch-Pagan-Godfrey $p = 0.060$). The log-linear specification satisfied the homoscedasticity assumption ($p = 0.667$), confirming that it is the appropriate functional form for our data and also provided a slight improvement in adjusted R^2 (0.92 vs. 0.89). Importantly, the coefficients for key variables (particularly body weight and body condition) remained significant and consistent in sign across both specifications.

In summary, the high adjusted R^2 reflects the nature of cattle as relatively homogeneous assets whose prices are determined by observable characteristics. The diagnostic results already reported in the Methods section, along with consistency across functional forms, suggest that overfitting is not a concern.

6) Specification of random parameters of the mixed logit models (Reviewer #2)

The bull size and temperament were defined as random parameters, as well as the cow's milk yield and calf vigor. A more detailed explanation of the rationale for the selection of these particular attributes should be provided. Furthermore, the authors should provide some discussion about the meaning and significance of the estimated standard deviations of the random parameters given the very small estimates reported.

Authors' Response:

We thank the reviewer for this important methodological observation. We appreciate the opportunity to explain our specification of random parameters and to discuss the interpretation of the estimated standard deviations.

The standard Multinomial Logit (MNL) model requires the Independence of Irrelevant Alternatives (IIA) assumption, which our data could not satisfy. To relax the IIA assumption and account for heterogeneity, we employed a mixed logit model with correlated random parameters. This model requires specifying random and fixed parameters. Following Hensher and Greene (2003), "*attributes that contributed most to overall model fit—based on Log Likelihood changes—were specified as random parameters, while others were modeled as fixed.*" For bulls, body size and temperament were specified as random; for cows, milk yield and calf vigor were specified as random.

The standard deviation of a random parameter indicates the spread of individual preferences around the mean coefficient. The standard deviations for body size (0.001), temperament (0.010), and milk yield (0.001) are very small, indicating broad consensus among farmers. For calf vigor, however, the standard deviation is somewhat larger (0.033) but remains modest.

In the revised manuscript, we have added the following text in the Discussion section (lines 494 to 499) for further clarification:

"The relatively small standard deviations indicate broad consensus among farmers on the importance of body size, temperament, and milk yield. While the standard deviation for calf vigor was somewhat larger, it remains modest. These findings suggest that a single breeding objective emphasizing these traits would serve the majority of smallholder farmers. This is particularly important in the context of smallholder breeding programs, where collective action and a large breeding population are required, making individualized breeding objectives impractical."

7) Calculation and interpretation of relative importance (Reviewer #2)

There is a need to provide a more detailed description of the method of determining relative importance from MWTP estimates. The authors should explain why MWTP values are suitable to calculate the relative importance of the various attributes and make sure the interpretation of the values is the same in the bull and cow model.

Authors' Response:

We thank the reviewer for this important methodological observation. We appreciate the opportunity to clarify our calculation of relative importance from MWTP estimates.

- **Method for Calculating MWTP and Relative Importance:**

As stated in the Methods section (lines 239 to 245), MWTP was calculated for each attribute using the following formula:

$$MWTP = -1\ X\ \left(\frac{Coefficient\ of\ the\ attribute}{Coefficient\ of\ price}\right)$$

The MWTP estimates the amount smallholders are willing to pay for a one-unit improvement in a specific attribute of a bull or cow.

Relative importance was then calculated from MWTP as follows:

$$Relative\ importance_k = \left(\frac{MWTP_k}{\sum MWTP\ for\ all\ attributes}\right) * 100$$

This provides the percentage contribution of each attribute to overall preference.

- **Why MWTP is suitable for calculating relative importance:** MWTP is suitable for calculating relative importance for the following reasons:
    - Common monetary scale: MWTP converts utility coefficients into a common monetary metric, allowing direct comparison across attributes with different units (e.g., body size vs. temperament vs. milk yield).

- Direct interpretation: MWTP reflects the actual trade-off farmers are willing to make between trait improvements and price—making relative importance estimates directly interpretable in economic terms. For example, as reported in the Results section (lines 296 to 297), "*smallholders were willing to pay an additional 17,520.8 ETB for a large body size and 16,384.1 ETB for a docile temperament relative to their baseline levels.*"
- We have included the following text in the Methods section of the revised manuscript (lines 248 to 251) for further clarification:
  "The MWTP is suitable for calculating relative importance because it converts utility coefficients into a common monetary scale, allowing direct comparison across attributes with different units. This approach was applied consistently to both the bull and cow models, ensuring that relative importance values are directly comparable."

In summary, MWTP was calculated using the standard formula, and relative importance was derived as the percentage contribution of each attribute's MWTP to the total. This method is suitable because MWTP provides a common monetary scale for comparison, and the same method was applied consistently to both bull and cow models. We are grateful to the reviewer for prompting this clarification.

8) Justification of attribute levels used in the DCE (Reviewer #2)

Strong empirical support may be needed for some of the attributed levels, especially milk production (>5 L/day vs. <2 L/day) and the resistance to disease (animals that "never get ill"). The authors should describe the selection of these thresholds and the extent to which they cover reasonable thresholds of variation under the production conditions used in the study area.

Authors' Response:

We thank the reviewer for this important observation. We appreciate the opportunity to explain how attribute levels were determined and to justify the specific thresholds used.

- **General approach to attribute level selection:** As stated in the Methods section of (lines 134 to 145), attribute levels were determined through Participatory Rural Appraisal (PRA), pairwise ranking with key informants, and consultations with researchers and livestock experts.
- **Justification for milk production thresholds** (>5 L/day vs. <2 L/day):The milk production thresholds were selected based on empirical data from Ethiopia:
  - Upper level (>5 L/day): Based on daily milk yield of crossbred cows (50–87.5% exotic blood level), reported at 5.7–6.9 L/day (Haile et al., 2008).
  - Lower level (<2 L/day): Based on average daily milk yield of unimproved indigenous cows, reported at approximately 1.5 L/day (Mekuriaw & Harris-Coble, 2021).

    Together, these thresholds capture the plausible range of variation from unimproved indigenous cattle to improved crossbred cattle under smallholder

conditions. We have added a footnote below Table 2 providing this empirical justification (lines 150 to 151).

- **Justification for disease resistance definition:** Disease resistance is already defined in operational and measurable terms in Table 2: The measurement is based on the frequency of visiting a veterinary clinic per year due to illness of a bull or cow. This provides a measurable and realistic range of variation under smallholder conditions.

In summary, attribute levels were selected through a rigorous process. Milk production thresholds are empirically grounded in Ethiopian production data, and disease resistance is defined operationally in Table 2. We are grateful to the reviewer for prompting this clarification.

9) There are no options for opting out (Reviewer #2)

In all choice tasks, none of the 384 respondents chose the opt-out option. This is a significant discovery which warrants further discussion. The authors must take into account the credibility of the alternatives, how the opt-out alternative was presented, or the understanding the respondents may have of the choice experiment.

Authors' response:

We thank the reviewer for this important observation. This issue was also raised by Reviewer #1 (Comment #3), and we have provided a comprehensive response there. To avoid redundancy, we summarize the key points below.

- **Contextual Justification:**

A zero or near-zero opt-out rate is uncommon in consumer-market DCEs, but it is behaviorally rational in the context of smallholder livestock breeding objectives. Previous studies in Ethiopia have reported similarly low opt-out rates: Abebe et al. (2020) reported <0.5% for sheep traits, and Kassie et al. (2009) reported 1.4% for indigenous cow traits. In our study, the opt-out was framed as "No genetic improvement or trait modification is needed for your herd relative to the presented options." Given the subsistence constraints of smallholder crop-livestock systems—where cattle are critical for food, income, and draft power—choosing the status quo was viewed as unrealistic and non-viable.

- **Credibility of alternatives:**

The traits included were identified through Participatory Rural Appraisal (PRA) and expert consultation. Levels were set based on empirical data from Ethiopia (e.g., milk yield thresholds: Haile et al., 2009; Mekuriaw & Harris-Coble, 2021). The alternatives presented realistic and meaningful improvements over the status quo.

- **Presentation of opt-out and respondent understanding:**

The opt-out alternative was explicitly framed to farmers as: "No genetic improvement or trait modification is needed for your herd relative to the presented options." Since the objective of the

study was to identify breeding preferences (traits to be genetically improved), framing the opt-out in this manner was highly appropriate.

Visual aids: Pictures and icons represented traits and levels; the opt-out was clearly labeled at the bottom of each choice card.

Enumerator training: All enumerators held a BSc in Animal Science and were trained to present all options fairly and transparently, without prompting or rushing.

Supervision: Field supervisors conducted random spot-checks to ensure all alternatives were presented transparently. The zero-frequency trend was uniform across all enumerators, ruling out isolated interviewer bias.

We have added a concise paragraph to the revised manuscript (lines 174 to 184) acknowledging the zero opt-out rate, explaining its behavioral rationality in the smallholder context, and noting that the opt-out was excluded from the final estimation matrix to ensure model convergence.

For the full detailed response and the exact manuscript text, please refer to our reply to Reviewer #1, Comment #4. We are grateful to the reviewer for prompting this clarification.

10) For breeding objective development implications will be discussed (Reviewer #2)

The manuscript suggests body size, milk yield and other characteristics as requirements for breeding objectives. The study doesn't, however, attempt to calculate genetic parameters, genetic correlations, or economic weights needed to develop a formal selection index. The authors should therefore temper some of the recommendations and provide some clarity that the results indicate priority traits and farmer/market preferences but are not an exhaustive economically optimized selection index.

Authors' Response:

We thank the reviewer for this important observation. We agree that our study identifies priority traits and farmer/market preferences but does not calculate genetic parameters, genetic correlations, or economic weights needed to develop a formal selection index. We have tempered our recommendations and clarified the scope of our findings.

- **Clarification of Scope and Logical Relationship:**

We would like to respectfully clarify the distinction between defining breeding objectives and developing a selection index:

  - Defining breeding objectives involves identifying the traits to be improved based on farmer preferences and market demand. This is a standalone, essential step in breeding program design. This is what we did.
  - Estimating genetic parameters and economic values must be done for specific traits—those identified through breeding objective definition. This is not part of our study.

- Developing a selection index (or breeding scheme) requires identifying breeding objective traits, estimating genetic and phenotypic parameters, and deriving economic values if more than one trait is considered. This is beyond the scope of our study.

Therefore, defining breeding objectives is logically prior to—and essential for—genetic parameter estimation and economic weight derivation. Our study provides the essential foundation: priority traits and farmer/market preferences that determine which traits should be evaluated in subsequent genetic and economic analyses.

We have revised words related to selection index and genetic correlations in the Discussion and Conclusion sections to make clear that our study identifies priority traits.

11) Overall Recommendation (Reviewer #2)

The manuscript is of scientific interest and may provide benefit in designing conservation-based cattle breeding programs. The above problems are mostly related to problems of clarity, interpretation and strengthening of methodological justification and do not reflect fundamental flaws in the study design. Readdressing these points would significantly enhance the rigor and clarity of the manuscript.

Authors' response:

We sincerely thank the reviewer for this encouraging assessment and for the constructive comments provided throughout the review. We are grateful that the reviewer considers the manuscript to be of scientific interest and of potential benefit for designing conservation-based cattle breeding programs. Point-by-point responses have been made to address each of the reviewer's comments. We believe these revisions have enhanced the rigor, clarity, and transparency of the manuscript, and we are grateful to the reviewer for the thoughtful engagement that made these improvements possible.

We have addressed each of the reviewer's comments in the point-by-point responses above. Key revisions include:

##

Once again, we thank the Editor and the reviewers for their thoughtful and constructive feedback. Their comments have been invaluable in strengthening our manuscript, and we truly appreciate the time and expertise they devoted to this review process.

Abiy Shenkut Abebe

Corresponding author
